# Dihydroartemisinin suppresses pancreatic cancer cells via a microRNA-mRNA regulatory network

**DOI:** 10.18632/oncotarget.11517

**Published:** 2016-08-23

**Authors:** Yilong Li, Yongwei Wang, Rui Kong, Dongbo Xue, Shangha Pan, Hua Chen, Bei Sun

**Affiliations:** ^1^ Department of Pancreatic and Biliary Surgery, The First Affiliated Hospital of Harbin Medical University, Harbin, 150001, China; ^2^ Department of General Surgery, The First Affiliated Hospital of Harbin Medical University, Harbin, 150001, China

**Keywords:** dihydroartemisinin, pancreatic cancer, microRNA, microRNA-mRNA regulatory network

## Abstract

Despite improvements in surgical procedures and chemotherapy, pancreatic cancer remains one of the most aggressive and fatal human malignancies, with a low 5-year survival rate of only 8%. Therefore, novel strategies for prevention and treatment are urgently needed. Here, we investigated the mechanisms underlying the anti-pancreatic cancer effects dihydroartemisinin (DHA). Microarray and systematic analysis showed that DHA suppressed proliferation, inhibited angiogenesis and promoted apoptosis in two different human pancreatic cancer cell lines, and that 5 DHA-regulated microRNAs and 11 of their target mRNAs were involved in these effects via 19 microRNA-mRNA interactions. Four of these microRNAs, 9 of the mRNAs and 17 of the interactions were experimentally verified. Furthermore, we found that the anti-pancreatic caner effects of DHA *in vivo* involved 4 microRNAs, 9 mRNAs and 17 microRNA-mRNA interactions. These results improve the understanding of the mechanisms by which DHA suppresses proliferation and angiogenesis and promotes apoptosis in pancreatic cancer cells and indicate that DHA, an effective antimalarial drug, might improve pancreatic cancer treatments.

## INTRODUCTION

Despite decades of effort to improve treatments, pancreatic cancer remains one of the most aggressive and deadly human tumors, with a five-year survival rate of only 8% [1]. Pancreatic cancer is currently the eighth and ninth leading cause of worldwide cancer-related death in men and women, respectively [2]. However, patients who are treated with multimodal therapy, including surgical resection, have a 5-year survival rate of more than 20% [3]. This unfavorable prognosis for pancreatic cancer is primarily due to the poor therapeutic effects of chemotherapy and radiotherapy, the aggressive biological behavior of pancreatic tumors, and late diagnoses. Alternative therapeutic strategies are urgently needed to more effectively treat this deadly disease.

MicroRNAs, a family of non-coding RNAs approximately 21-23 nucleotides in length, regulate gene expression post-transcriptionally by targeting the 3′-untranslated regions (3′-UTR) of target genes through complementary base pairing, and play significant roles in a variety of biological processes, particularly in cancer cells [4, 5]. MicroRNAs negatively regulate their targets by degrading mRNA or inhibiting its translation. microRNAs may target more than 30% of human genes [6] and regulate survival, apoptosis, proliferation, angiogenesis, invasion, metastasis, and chemotherapy resistance during the initiation and progression of human cancers[7–9]. Therefore, strategies that modulate microRNAs to regulate tumor amplification, apoptosis, metastasis, and invasion may provide promising novel treatments for pancreatic cancer.

Dihydroartemisinin (DHA), the main active metabolite of the artemisinin derivative antimalarial drugs, has anticancer effects in pancreatic cancer [10], lung cancer [11], breast cancer [12], cervical cancer [13], and glioma [14], and inhibits angiogenesis in cancer [15, 16]. We previously found that DHA induces G0/G1 cell cycle arrest [10] and apoptosis [17], inhibits growth [17] and angiogenesis [15], and increases sensitivity to chemotherapy [18, 19] in pancreatic cancer. However, the details of the mechanisms underlying DHA's anti-pancreatic cancer effects have not been fully described; here, we further investigated these mechanisms.

Many experiments have confirmed that microRNAs play important roles as either promotors or inhibitors of cancer [20–22]. However, few experiments have demonstrated that drugs directly influence the ability of microRNAs to modulate their target genes and thus affect survival, apoptosis, proliferation, angiogenesis, invasion, metastasis, and chemotherapy resistance in cancer. In this study, we examined differentially expressed microRNAs from microarray profiles, confirmed microRNA-mRNA interactions using experimentally validated databases, and conducted a functional enrichment analysis of pathways from the Kyoto Encyclopedia of Genes and Genomes (KEGG) using quantitative real-time polymerase chain reactions and western blots, to investigate the mechanisms underlying DHA's anti-pancreatic cancer effects.

## RESULTS

### Aberrant microRNA expression profiles in PANC-1 and BxPC-3 pancreatic cancer cells

**Figure 1:**
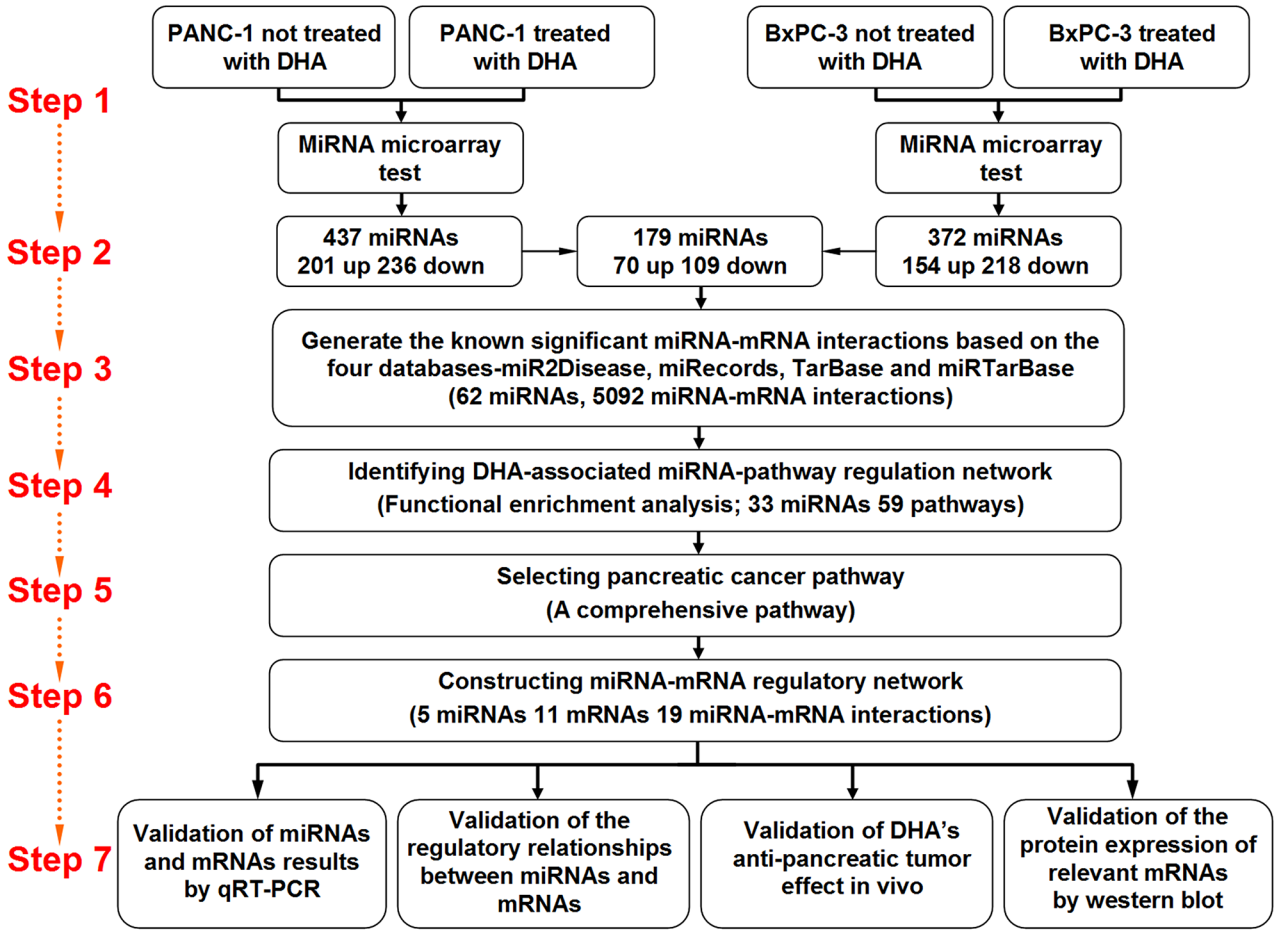
The multi-step approach adopted to identify microRNAs differentially expressed after DHA treatment and their target mRNAs in two pancreatic cancer cell lines

MicroRNA expression profiles in DHA-treated and control PANC-1 and BxPC-3 pancreatic cancer cells were examined using a microRNA microarray. microRNA expression results were normalized using median normalization and used to screen for differentially expressed microRNAs. Differentially expressed microRNAs were identified via fold-change filtering between the two groups in each cell line; a fold-change ≥ 1.5 identified microRNAs as up- or down-regulated. The identified microRNAs were then filtered between the two cell lines such that microRNAs for which expression was similarly up- or down-regulated in both PANC-1 and BxPC-3 cells were considered differentially expressed microRNAs. Seventy microRNAs that were up-regulated and 109 that were down-regulated (fold change ≥ 1.5) in both PANC-1 and BxPC-3 pancreatic cancer cells were identified using these filters (Figure 1).

### Identification of DHA-associated microRNA-mRNA interaction networks

**Figure 2:**
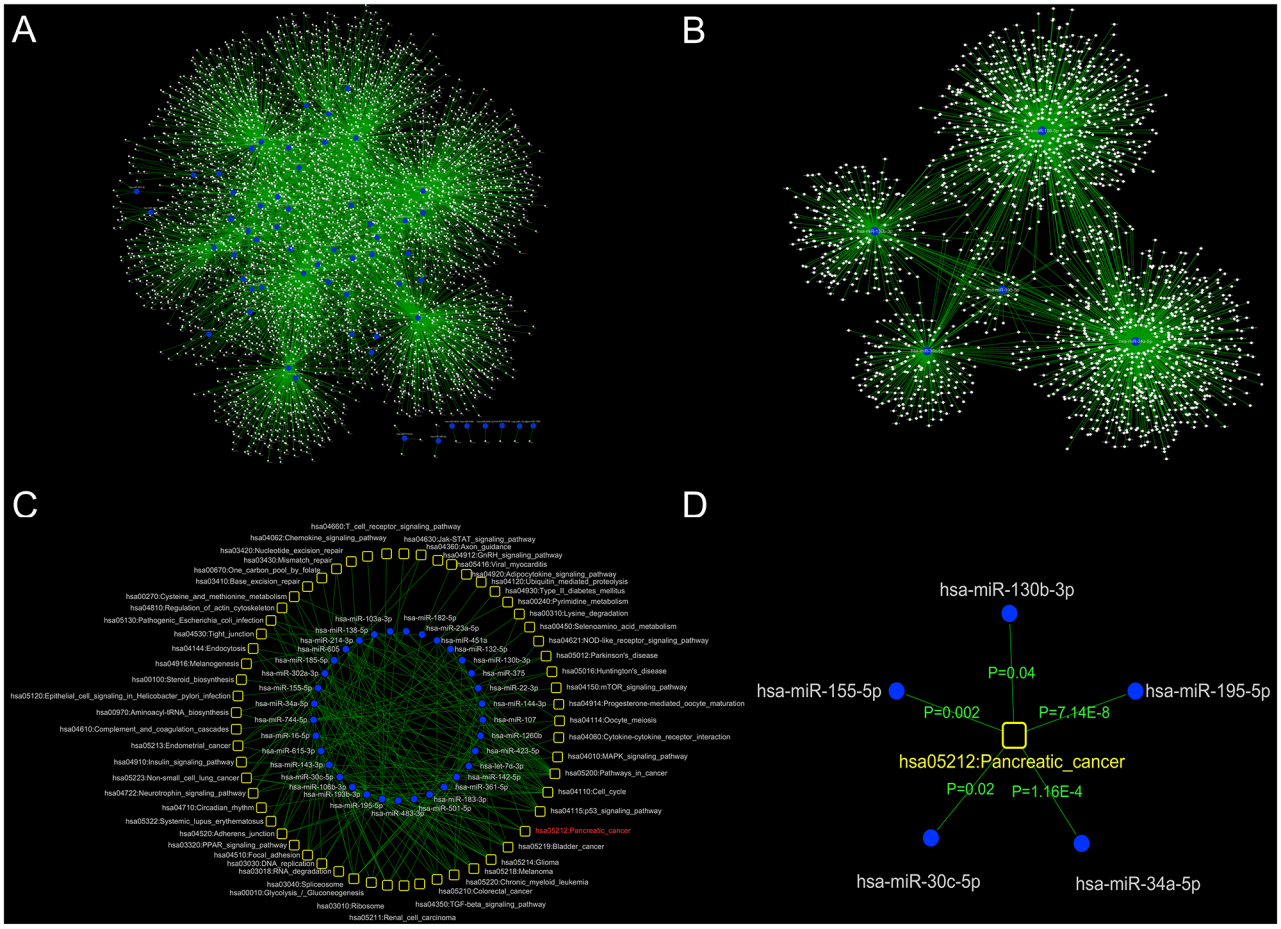
Global view of the DHA-associated microRNA-mRNA regulation and DHA-associated microRNA-pathway regulatory networks **A.** To identify microRNAs and their targets (mRNAs) that were regulated by DHA, four experimentally verified microRNA-mRNA interaction databases were examined. 5,092 microRNA-mRNA pairs were identified that involved 62 of the differentially expressed microRNAs. **B.** The selected microRNAs and their targets (mRNAs) were used to construct the DHA-regulated microRNA-mRNA regulatory network. **C.** A global view of the DHA-associated microRNA-pathway regulatory network. Functional enrichment analysis was performed based on the microRNA-mRNA interaction network to identify biological functions of microRNAs that were regulated by DHA. Ultimately, 204 microRNA-pathway interactions were constructed between 33 microRNAs and 59 pathways. **D.** The microRNAs were significantly associated with the KEGG pathway database pancreatic cancer pathway (*p*<0.05).

Four high-confidence microRNA-mRNA interaction databases (miR2Disease, miRecords, TarBase and miRTarBase) were used together to screen the experimentally identified differentially expressed microRNAs and their targets in DHA-treated cells. As shown in Figure 2A, 5,092 microRNA-mRNA interactions were identified between 62 of the 179 differentially expressed microRNAs (117 had no significant interactions) and 4,362 mRNAs. The node degree distribution of this network revealed a scale-free structure, with R^2^=0.86. These results indicate that the DHA-associated microRNA-mRNA interaction network is well-characterized by a core set of post-transcriptional regulatory relationships that are distinct from those of randomly generated networks (25800746).

### Identification of DHA-associated microRNA-pathway regulation networks

To determine which molecular pathways are regulated by these differentially expressed microRNAs in response to DHA treatment, a functional enrichment analysis was performed based on the microRNA-mRNA interaction network. Neighbor mRNA nodes in the network for each microRNA were used as input gene sets to examine relationships with hundreds of KEGG pathways. The hypergeometric test was used to identify significantly enriched pathways (*p*<0.05). As shown in Figure 2C, 204 microRNA-pathway interactions were identified, involving 33 microRNAs and 59 pathways. In the microRNA-pathway interaction network, several important cancer-related pathway categories, such as “pathways in cancer,” “cell cycle,” “P53 signaling,” and “pancreatic cancer pathways,” were connected to more microRNA nodes than others. These observations indicate that cancer risk-related pathways tend to be hub nodes in the microRNA-pathway interaction network. Cytoscape software (version 3.2.1) (14597658) was used to construct and illustrate the network.

### Characteristics of microRNAs that regulate the pancreatic cancer pathway

**Figure 3:**
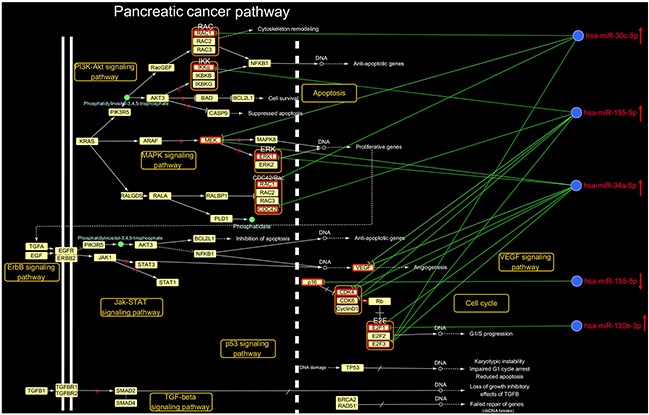
Details regarding the microRNA-mRNA regulatory network in the pancreatic cancer pathway In the KEGG PATHWAY database pancreatic cancer pathway (http://www.genome.jp/kegg/pathway.html), 11 key mRNAs were regulated by 5 microRNAs that were up-regulated by DHA. ↑ indicates up-regulation by DHA, and ↓ indicates down-regulation by DHA.

To investigate the mechanisms by which DHA exerts anti-pancreatic cancer effects, we examined the pancreatic cancer pathway identified in the DHA-associated microRNA-pathway regulation network. The comprehensive pancreatic cancer pathway includes the PI3K-Akt, MAPK, ErbB, Jak-STAT, VEGF, p53, and TGF-β signaling pathways, as well as cell cycle and apoptosis pathways. A detailed examination of the microRNA target sites in the pancreatic pathway map is shown in Figure 3. Eleven key pancreatic cancer pathway mRNAs were regulated by 5 microRNAs. For example, miR-34a-5p regulated Cdk4, and miR-195-5p regulated CDC42; both miR-34a-5p and miR-195-5p were up-regulated by DHA and co-regulated Cdk6, VEGF, E2F3, and Cdk4; the up-regulated microRNA miR-30c-5p regulated Rac1, and co-regulated MEK1 with miR-34a-5p and E2F3 with miR-34a-5p and miR-195-5p; the up-regulated microRNA miR-130b-3p co-regulated E2F1 with miR-34a-5p; and the down-regulated microRNA miR-155-5p regulated p16.

### Confirmation of microRNA-target mRNA regulatory relationships with western blots

To assess the regulatory relationships between the microRNAs and target mRNAs identified via microarray and systematic analysis, we next transfected PANC-1 and BxPC-3 cells with miR-34a-5p, miR-195-5p, miR-30c-5p, or miR-130b-3p or their inhibitors and examined the protein levels of their target mRNAs, including Cdk4, Cdk6, VEGF, IKKα, MEK1, E2F3, Rac1, E2F1, and CDC42 in western blots.

**Figure 4:**
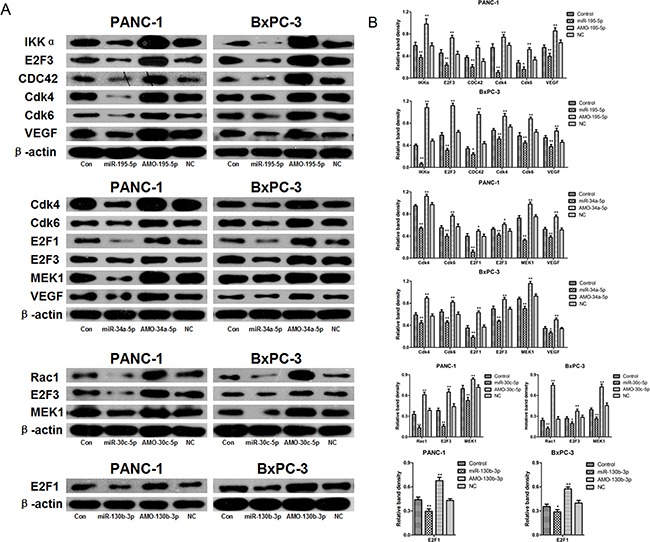
Confirmation of regulatory relationships between microRNAs and target mRNAs by western blot **A.** PANC-1 and BxPC-3 cells were transfected with miR-34a-5p, miR-195-5p, miR-30c-5p, miR-130b-3p, AMO-34a-5p, AMO-195-5p, AMO-30c-5p, AMO-130b-3p, or negative control miRNA. Cdk4, Cdk6, VEGF, IKKα, MEK1, E2F3, Rac1, E2F1, and CDC42 protein levels were detected by western blot. β-actin was used as a protein loading control. **B.** The density of each band was measured and compared to β-actin. *p<0.05, **p<0.01 compared to the control.

As shown in Figure 4, miR-30c-5p overexpression reduced Rac1, MEK1, and E2F3 levels in both PANC-1 and BxPC-3 cells. Conversely, inhibition of endogenous miR-30c-5p increased Rac1, MEK1, and E2F3 levels compared to the control group. Similarly, miR-195-5p overexpression reduced, and inhibition of endogenous miR-195-5p increased, IKKα, CDC42, VEGF, Cdk4, Cdk6, and E2F3 levels in both PANC-1 and BxPC-3 cells compared to the control group. miR-34a-5p overexpression reduced, and inhibition of endogenous miR-34a-5p increased, MEK1, VEGF, Cdk4, Cdk6, E2F1, and E2F3 levels in both PANC-1 and BxPC-3 cells compared to the control group. Finally, miR-130b-3p overexpression reduced, and inhibition of endogenous miR-130b-3p increased, E2F1 at levels in both PANC-1 and BxPC-3 cells compared to the control group. We did not confirm the regulatory relationships between miR-155-5p and p16 and miR-34a-5p and ERK1.

### Confirmation of microRNA-target mRNA regulatory relationships with qRT-PCR

To confirm the results of microarray experiments and mRNA data obtained from the experimentally validated databases, all of the microRNAs (miR-34a-5p, miR-195-5p, miR-30c-5p, and miR-130b-3p) that were up-regulated by DHA, suppressed growth and angiogenesis, and promoted apoptosis in pancreatic cancer cells, and their target mRNAs (Cdk4, Cdk6, VEGF, IKKα, MEK1, E2F3, Rac1, E2F1, and CDC42), were analyzed with qRT-PCR.

**Figure 5:**
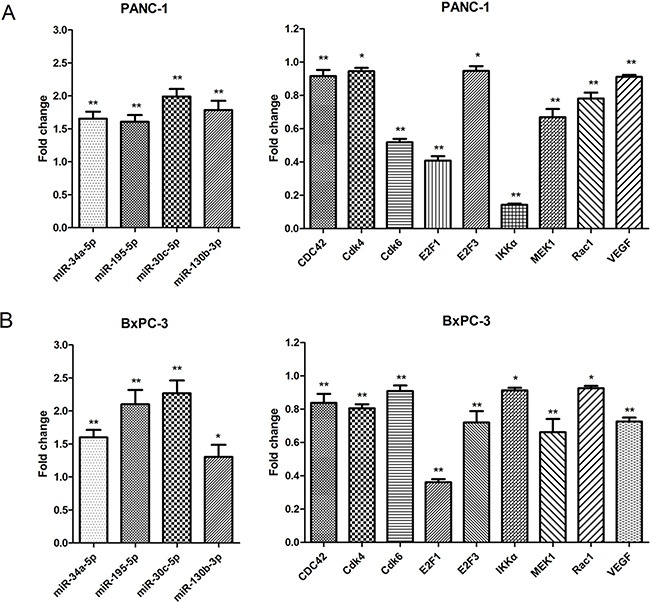
Confirmation of microarray and systematic analysis results by qRT-PCR Confirmation of microarray and systematic analysis results by qRT-PCR. Differential expression of the 4 microRNAs and their 9 mRNA targets were consistent with microarray tests and systematic analysis data. **A.** Differential expression of the 4 microRNAs and their 9 mRNA targets in the PANC-1 cell line. *p<0.05, **p<0.01 compared to the control. **B.** Differential expression of the 4 microRNAs and their 9 mRNA targets in the BxPC-3 cell line. *p<0.05, **p<0.01, compared to the control.

As shown in Figure 5, miR-34a-5p, miR-195-5p, miR-30c-5p, and miR-130b-3p were up-regulated in DHA-treated PANC-1 and BxPC-3 cells compared to vehicle-treated controls, confirming the microarray results. Additionally, Cdk4, Cdk6, VEGF, IKKα, MEK1, E2F3, Rac1, E2F1, and CDC42 mRNA expression were down-regulated in DHA-treated PANC-1 and BxPC-3 cells compared to vehicle-treated controls, confirming the microarray results and systematic database analysis (Figure 5). Similar results were also obtained in SW1990 and CFPAC-1 cells (Supplementary Figure S1). All of the microRNAs (miR-34a-5p, miR-195-5p, miR-30c-5p, and miR-130b-3p) were also analyzed with qRT-PCR in HPDE6-C7 cell line (Supplementary Figure S2).

### Confirmation of relevant mRNA protein levels with western blots

To determine protein levels of the mRNAs modulated by DHA via microRNAs, we next examined Cdk4, Cdk6, VEGF, IKKα, MEK1, E2F3, Rac1, E2F1, and CDC42 protein levels, which affect growth, inhibit angiogenesis, and promote apoptosis in DHA-treated and vehicle-treated pancreatic cancer cells.

**Figure 6:**
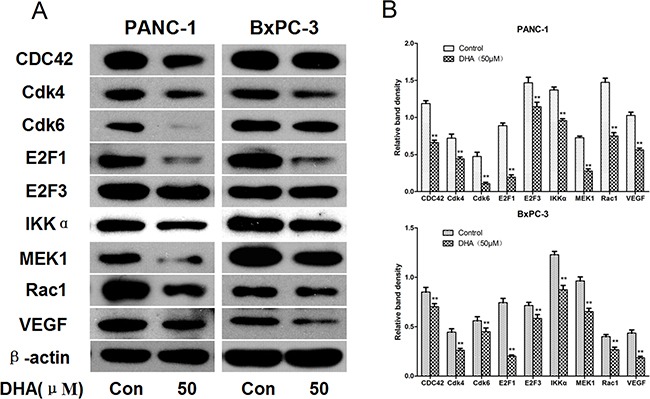
Confirmation of protein levels of the relevant mRNAs by western blot **A.** PANC-1 and BxPC-3 pancreatic cancer cells were treated with DHA (50 μM) or vehicle for 72 h, and the protein extracts were measured by western blot. β-actin was used as a protein loading control. **B.** The density of each band was measured and compared to β-actin. *p<0.05, **p<0.01, compared to the control.

Western blots revealed that Cdk4, Cdk6, VEGF, IKKα, MEK1, E2F3, Rac1, E2F1, and CDC42 levels decreased in PANC-1 and BxPC-3 cells that were treated with 50 μM DHA for 72 h (Figure 6). Similar results were obtained in SW1990 and CFPAC-1 cells (Supplementary Figure S3).

### microRNAs identified by microarrays and systematic analysis contribute to the anti-pancreatic cancer effects of DHA

To confirm that the microRNAs identified by microarray and systematic analysis contribute to the anti-pancreatic cancer effects of DHA, western blots were used to measure target mRNA protein levels in the presence of microRNA inhibitors. As shown in Supplementary Figure S4, DHA reduced Cdk4, Cdk6, VEGF, IKKα, and Rac1 levels compared to the control group. However, transfection of AMO-195-5p, AMO-34a-5p, or AMO-30c-5p reversed this DHA-induced inhibition compared to the group treated with DHA alone. These results indicate that the anti-pancreatic cancer effects of DHA were mediated at least partly by miR-195-5p, mir-34a-5p, and mir-30c-5p.

### DHA suppresses growth, inhibits angiogenesis, and promotes apoptosis *in vivo*

**Figure 7:**
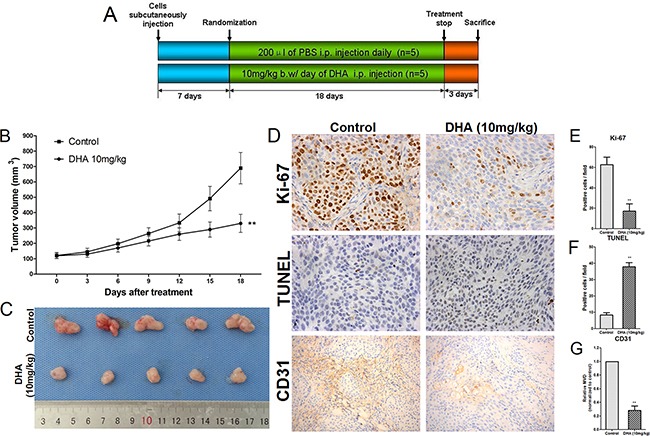
DHA decreases proliferation and angiogenesis and increases apoptosis in pancreatic cancer cells *in vivo* **A.** Schematic representation of the experimental protocol described in the Methods. **B.** Tumors were measured using Vernier calipers and volumes were calculated using the formula V=(π/6)×(larger diameter)×(smaller diameter)^2^. **C.** Mice were sacrificed and tumors were excised at the end of treatment. **D.** Cell proliferation was measured by immunohistochemical analysis of Ki-67, apoptotic cells were measured by TUNEL staining, and microvessel density was measured by CD31 immunohistochemistry in tumor tissues. **E.** Ki-67-positive cells were counted to estimate the proliferation index. **F.** TUNEL-positive cells were counted to estimate the apoptosis index. **G.** CD31-stained microvessels were counted to estimate microvessel density. *p<0.05, **p<0.01 compared to the control.

To examine the anti-tumor effects of DHA *in vivo*, we established a xenograft pancreatic cancer model in nude mice that did or did not receive 18 days of DHA treatment (Figure 7A). Tumor sizes were measured every three days, and volumes were estimated according to the formula V=(π/6)×(larger diameter)×(smaller diameter)^2^ (Figure 7B). DHA treatment decreased tumor volumes over time compared to the control group. Mice were sacrificed and tumors excised after treatment (Figure 7C).

To evaluate the anti-proliferation, anti-angiogenesis, and apoptotic effects of DHA in tumor tissues, Ki-67 (a cell proliferation marker) and CD31 (a microvessel density marker) levels and TUNEL staining were examined using immunohistochemistry. As shown in Figure 7D, tumors from DHA-treated mice had fewer Ki-67-positive cells than those from the vehicle-treated group. Similarly, DHA treatment reduced microvessel density compared to the vehicle-treated group (Figure 7D). DHA treatment also increased the percentage of TUNEL positive cells compared to the vehicle-treated group (Figure 7D).

**Figure 8:**
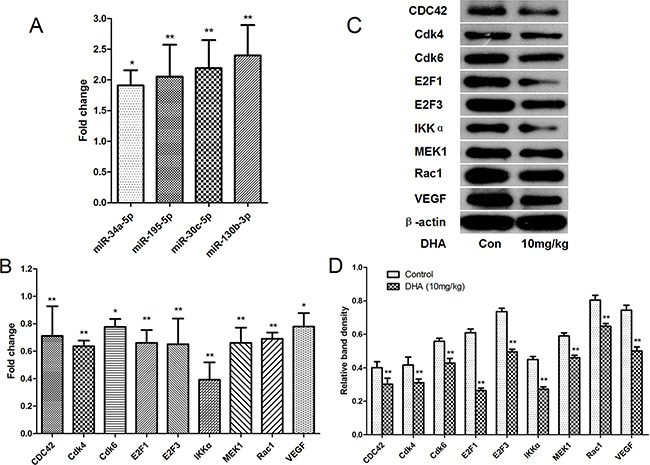
Confirmation of microRNAs and target mRNAs identified via microarray and systematic analysis by qRT-PCR and western blot in pancreatic tumor tissues **A.** Differential expression of the 4 microRNAs in pancreatic tumor tissues. **B.** Differential expression of the 9 mRNA targets in pancreatic tumor tissues. **C.** Cdk4, Cdk6, VEGF, IKKα, MEK1, E2F3, Rac1, E2F1, and CDC42 protein levels in pancreatic cancer tissues were detected by western blot. β-actin was used as a protein loading control **D.** The density of each band was measured and compared to β-actin. *p<0.05, **p<0.01 compared to the control.

To analyze the mechanism by which DHA suppresses growth, inhibits angiogenesis, and promotes apoptosis in tumor tissues, expression of the microRNAs (miR-34a-5p, miR-195-5p, miR-30c-5p, and miR-130b-3p) that were up-regulated by DHA and their target mRNAs (Cdk4, Cdk6, VEGF, IKKα, MEK1, E2F3, Rac1, E2F1, and CDC42) were analyzed using qRT-PCR. As shown in Figure 8A, miR-34a-5p, miR-195-5p, miR-30c-5p, and miR-130b-3p were up-regulated in the DHA-treated group compared to the vehicle-treated group, confirming the microarray results *in vivo*. Additionally, Cdk4, Cdk6, VEGF, IKKα, MEK1, E2F3, Rac1, E2F1, and CDC42 mRNA expression were down-regulated in the DHA-treated group compared to the vehicle-treated group, experimentally confirming the microarray results and systematic database analysis *in vivo* (Figure 8B).

We then measured protein levels of Cdk4, Cdk6, VEGF, IKKα, MEK1, E2F3, Rac1, E2F1, and CDC42, the mRNAs of which were down-regulated by DHA via microRNAs, and which affect growth, inhibit angiogenesis, and promote apoptosis. Western blots showed that tumors from mice treated with 10 mg/kg DHA for 18 days had decreased Cdk4, Cdk6, VEGF, IKKα, MEK1, E2F3, Rac1, E2F1, and CDC42 levels compared to control group mice (Figure 8C).

## DISCUSSION

The American Cancer Society estimates that approximately 53,070 Americans will be diagnosed with pancreatic cancer in 2016. Despite significant efforts to improve treatments, 41,780 Americans will die from pancreatic cancer, which has a five-year survival rate of only 8% [1]. Gemcitabine, a broad spectrum drug used to treat solid tumors, is well-tolerated in pancreatic cancer patients. However, patients who show initial sensitivity to gemcitabine chemotherapy rapidly acquire resistance, and the efficacy of gemcitabine in treating pancreatic cancer remains at only 20-30% [9, 23]. Therefore, novel and effective chemotherapeutic agents for the treatment of pancreatic cancer are urgently needed.

DHA is an effective anti-malarial drug, and many studies have revealed that DHA also has anticancer effects in a variety of cancers. In a previous study, we found that DHA suppressed pancreatic cancer cell growth both *in vitro* and *in vivo* [17]. We also found that DHA down-regulated cdks and cyclins, such as Cdk4, Cdk6, and cyclin E, which play essential roles in the regulation of cell cycle progression; DHA thus increased G0/G1 cell cycle arrest in pancreatic cancer cells. More importantly, DHA inhibited the DNA-binding activity of NF-κB in pancreatic cancer cells [10]. Furthermore, DHA increased the anti-pancreatic cancer effects of gemcitabine by inactivating NF-κB both *in vitro* and *in vivo* [18] and suppressed angiogenesis by regulating the NF-κB pathway [15]. Finally, we found that DHA enhanced Apo2L/TRAIL-mediated apoptosis via ROS-mediated up-regulation of death receptor 5 [19]. In summary, we have demonstrated that DHA, in addition to curing malaria, is a promising chemotherapeutic agent for treating pancreatic cancer. However, the mechanisms underlying the anti-pancreatic cancer effects of DHA are not fully understood. Therefore, we investigated the mechanisms by which DHA exerts anti-pancreatic cancer effects using microarray profiles and systematic analyses.

To our knowledge, this is the first study to examine differences in microRNA expression after DHA treatment using microarrays and systematically analyze DHA-associated microRNA-mRNA interaction networks to identify the mechanisms by which DHA exerts its anti-cancer effects. Surprisingly, we found that four crucial microRNAs (miR-34a-5p, miR-195-5p, miR-30c-5p, and miR-130b-3p) regulated the expression of many mRNAs (Cdk4, Cdk6, VEGF, IKKα, MEK1, E2F3, Rac1, E2F1, ERK1, and CDC42) and their proteins, and thus were crucial to the anti-pancreatic cancer effects of DHA.

Cdk4, Cdk6, E2F3, and E2F1 play key roles in the regulation of cell cycle progression in pancreatic cancer. Down-regulation of Cdk4, Cdk6, E2F3, and E2F1 expression increases G0/G1 cell cycle arrest in pancreatic cancer cells [24, 25]. Here, we found that DHA treatment up-regulated miR-34a-5p, miR-195-5p, miR-130b-3p, and miR-30c-5p expression and down-regulated the expression of the target mRNAs Cdk4, Cdk6, E2F3, and E2F1, respectively; DHA treatment also decreased protein levels translated from these mRNAs. VEGF plays a key role in angiogenesis [26], and down-regulation of VEGF expression suppresses angiogenesis in pancreatic cancer. Here, we found that DHA treatment down-regulated VEGF mRNA expression and protein levels by up-regulating the expression of miR-34a-5p and miR-195-5p.

The Ras-Raf-MEK-ERK signalling pathway, which is one of best-characterized kinase cascades in cancer cell biology, influences various processes in tumors, including cancer cell survival, proliferation, migration, and differentiation [27]. MEK1 and ERK1 play key roles in the Ras-Raf-MEK-ERK signalling pathway [28]. In this study, DHA treatment up-regulated miR-34a-5p and miR-30c-5p and down-regulated MEK1 mRNA expression and protein levels, which both microRNAs target. These results indicate that the Ras-Raf-MEK-ERK signalling pathway is involved in the anti-pancreatic cancer effects of DHA. CDC42, a Rho family GTPase, also plays an important role in various cancers [29, 30] and promotes proliferation and metastasis [31, 32]. Here, the DHA-induced increase in miR-195-5p down-regulated CDC42 expression, likely contributing to the ability of DHA to inhibit proliferation and suppress metastasis. Rac1, another Rho family GTPase, is also involved in various cancers, and down-regulation of Rac1 expression inhibits proliferation, viability, and migration in pancreatic cancer cells [33]. Here, DHA-induced, miR-30c-5p-mediated down-regulation of Rac1 expression might be another novel mechanism by which DHA inhibits cancer cell proliferation, viability, and migration.

In a previous study, we found that, although total NF-κB levels did not change, DHA treatment down-regulated nuclear NF-κB levels in a dose-dependent manner [10]. Therefore, DHA prevented the transfer of NF-κB into the nucleus, and down-regulated gene products downstream of NF-κB, thus promoting apoptosis, suppressing growth, inhibiting angiogenesis, and increasing sensitivity to chemotherapy in pancreatic cancer cells. However, the mechanisms by which DHA prevents transport of NF-κB into the nucleus are unknown. Surprisingly, in the present study, DHA treatment up-regulated miR-195-5p, which down-regulated IKKα mRNA expression. IKK phosphorylates and degrades IκB in a ubiquitination-dependent manner; this prevents the translocation of the RelA(P65) and NF-κB1(P50) dimer, which is sequestered in the cytoplasm in its inactive state via interaction with the endogenous inhibitor IκB [34, 35]. This mechanism may explain the ability of DHA to inhibit the DNA-binding activity of NF-κB in pancreatic cancer cells.

In summary, we found that the DHA-induced microRNA-mRNA regulatory network suppressed growth, inhibited angiogenesis, and promoted apoptosis in pancreatic cancer. Our results provide mechanistic evidence that the anti-proliferative, pro-apoptotic, and anti-angiogenesis effects of DHA were associated with the up-regulation of miR-34a-5p, miR-195-5p, miR-30c-5, and miR-130b-3p. While some of these results confirmed our previous findings, most of them identified novel mechanisms underlying the anti-pancreatic cancer effects of DHA, which may be effective not only in treating malaria but also as a chemotherapeutic drug for treating pancreatic cancer.

## MATERIALS AND METHODS

### Strategy

As shown in Figure 1, a multi-step approach was used to identify differentially expressed microRNAs that were up-regulated by DHA and, in turn, the mRNAs regulated by these miRNAs, in two pancreatic cancer cell lines. First, PANC-1 and BxPC-3 pancreatic cancer cells were treated with DHA or vehicle, and the microRNAs that were differentially expressed between the treatment groups were identified using microarrays. 179 differentially expressed microRNAs were identified using two standards: first, the microRNAs were up- or down-regulated with a fold-change in expression ≥ 1.5; second, the microRNAs were differentially expressed similarly in both PANC-1 and BxPC-3 cell lines. Next, microRNA-mRNA interactions were identified using the miR2Disease, miRecords, TarBase, and miRTarBase databases; only microRNAs with confirmed target relationships in these databases were examined further. Based on the microRNA-mRNA interaction data, a DHA-associated microRNA-pathway regulatory network was identified, and the “pancreatic cancer pathway” specifically was selected for further investigation. Five microRNAs, 11 mRNAs, and 19 microRNA-mRNA interactions identified in the pancreatic cancer pathway were investigated to determine their roles in the mechanisms underlying the anti-pancreatic cancer effects of DHA. The differentially expressed microRNAs and mRNAs identified in this way were measured *in vitro* and *in vivo* using qRT-PCR, and levels of the protein products of these mRNAs were measured *in vitro* and *in vivo* using western blots. ThemicroRNA-target mRNA regulatory relationships selected by microarray and systematic analysis were confirmed with western blots in vitro. Finally, the anti-tumor effects of DHA were examined *in vivo* by measuring tumor sizes and via immunohistochemistry.

### Materials

Dihydroartemisinin (DHA) was purchased from Sigma-Aldrich, Inc. (St. Louis, MO, USA), dissolved in DMSO to make a 1000 mM stock solution, and stored at 4°C. The following antibodies were used: β-actin, Cdk4, Cdk6 (Santa Cruz Biotechnology, Carlsbad, CA, USA), VEGF (Abcam Inc., Cambridge, MA, USA), IKKα, MEK1 (Cell Signalling Technology, Inc., Danvers, MA, USA), E2F3, Rac1, E2F1, and CDC42 (Proteintech, Chicago, IL, USA).

### Cell culture

The human pancreatic cancer cell lines PANC-1, BxPC-3, SW1990, and CFPAC-1 were obtained from the American Type Culture Collection (Rockville, USA) and were cultured in DMEM or RPMI 1640 medium supplemented with fetal bovine serum (10%), penicillin (100 U/mL), and streptomycin (100 mg/mL) (Irvine Scientific, Irvine, CA). All cells were maintained at 37°C in humidified air with 5% CO_2_. All reagents were from HyClone China Ltd. (China). Mycoplasma contamination was tested using the Mycoplasma Stain Assay Kit (Beyotime Institute of Biotechnology, Beijing, China); none of the cell cultures were contaminated with mycoplasma.

### Treatment of cells

Subconfluent cells (60-70%) were treated with 50 μM DHA in DMSO in complete cell culture medium; control group cells were treated with 0.1% DMSO. Subsequent experiments were repeated three times.

### RNA extraction

Total RNA was extracted and isolated using TRIzol reagent (Invitrogen Life Technologies) according to the manufacturer's instructions. RNA quantity and quality were measured using a NanoDrop ND-2000 spectrophotometer. Acceptable OD A260/A280 ratios were between 1.8 and 2.1 (the target is approximately 2.0 for pure RNA), and OD A260/A230 ratios were greater than 1.8. RNA integrity was assessed by standard denaturing agarose gel electrophoresis.

### MicroRNA microarray procedure

After RNA was isolated from the samples, the miRCURY™ Hy3™/Hy5™ Power labeling kit (Exiqon, Vedbaek, Denmark) was used to label microRNAs according to the manufacturer's guidelines. One microgram of each sample was 3′-end-labeled with the Hy3^TM^ fluorescent label using T4 RNA ligase and the following procedure. RNA was suspended in 2.0 μL of water and combined with 1.0 μL of CIP buffer and CIP (Exiqon). The mixture was incubated for 30 min at 37°C, and the reaction was terminated by incubation for 5 min at 95°C. Then, 3.0 μL of labeling buffer, 1.5 μL of the fluorescent label (Hy3^TM^), 2.0 μL of DMSO, and 2.0 μL of labeling enzyme were added to the mixture. The labeling reaction was incubated for 1 h at 16°C and terminated by incubation for 15 min at 65°C. The Hy3^TM^-labeled samples were then hybridized on the miRCURY^TM^ LNA Array (v.18.0) (Exiqon) according to the array manual. The entire samples (Hy3^TM^-labeled samples in 25 μL of hybridization buffer) were denatured for 2 min at 95°C, incubated on ice for 2 min, and then hybridized to the microarray for 16-20 h at 56°C in a 12-Bay Hybridization System (Nimblegen Systems, Inc., Madison, WI, USA), which provides active mixing action at a constant incubation temperature to improve hybridization uniformity and enhance signals. Following hybridization, the slides were washed several times using the Wash buffer kit (Exiqon) and dried by centrifugation for 5 min at 400 rpm. The slides were then scanned using the Axon GenePix 4000B microarray scanner (Axon Instruments, Foster City, CA). The scanned images were imported into GenePix Pro 6.0 software (Axon) for grid alignment and data extraction. Replicate microRNAs were averaged, and microRNAs with intensities ≥30 in all samples were used to calculate the normalization factor. The expression data were normalized using median normalization. After normalization, differentially expressed microRNAs were identified through fold-change filtering. Hierarchical clustering was performed using MEV software (v4.6, TIGR).

### MicroRNA-mRNA interaction network

The experimentally verified miR2Disease (18927107), miRecords (18996891), TarBase (22135297), and miRTarBase (24304892) databases were used to identify microRNA-mRNA interaction networks; 43,260 non-redundant, validated microRNA-mRNA pairs were identified. Among these interactions, 5,092 microRNA-mRNA pairs were associated with 62 of the 179 differentially expressed microRNAs (there were no associations for the remaining 107) and incorporated into the microRNA-mRNA interaction network. Cytoscape software (version 3.2.1) (14597658) was used to construct and illustrate the network.

### Functional enrichment analysis

A hypergeometric test was used to calculate the significance of the enriched genes in KEGG pathways. If the whole genome had a total of N genes, of which K were involved in the biological pathway under investigation, and a total of M microRNA target genes, of which x were involved in the same pathway, were being analyzed, then the *p* value for the enrichment of that pathway was calculated as follows:

$$P=1-\sum_{t=0}^{x}\frac{\left(\begin{array}{l} K\\ t \end{array}\right)\left(\begin{array}{c} N-K\\ M-t \end{array}\right)}{\left(\begin{array}{c} N\\ M \end{array}\right)}$$

The pathways with *p*<0.05 were considered significantly enriched.

### Quantitative real-time polymerase chain reaction (qRT-PCR)

Total RNA was extracted from DHA-treated and vehicle-treated PANC-1 and BxPC-3 pancreatic cancer cells using the Ultrapure RNA Kit (CWBio Co., Ltd.) according to the manufacturer's instructions. The first-strand cDNA was synthesized using the HiFi-MMLV cDNA Kit (CWBio Co., Ltd.) for mRNA and using the miRNA cDNA Kit (CWBio Co., Ltd.) for miRNA. Real-time PCR was performed with the UltraSYBR Mixture (with ROX) (CWBio Co., Ltd.) for mRNA and the miRNA Real-Time PCR Assay kit for miRNA (CWBio Co., Ltd.) to quantify relative mRNA and miRNA expression. The reactions were run on a 7500 FAST Real-Time PCR System (Applied Biosystems). The PCR primer pairs are listed in Table 1. The reverse primers for miRNA were provided in the miRNA Real-Time PCR Assay kit. The relative expression of each gene was calculated using the comparative fold-change method (2^−ΔΔct^). β-actin and U6 were used as internal controls for mRNA and microRNA expression, respectively.

**Table 1:** Primers pairs for real-time PCR

Gene name	Forward primer (5′-3′)	Reverse primer (5′-3′)
Cdk6	AAAATCTTGGACGTGATTGGACTC	AGGTCCTGGAAGTATGGGTGAG
IKKα	GAGAGGAGGACCTGTTGACCTTACT	TTCCAGTTTCACGCTCAATACGA
VEGF	CAGATTATGCGGATCAAACCTCACC	CACAGGGAACGCTCCAGGACTTAT
E2F3	AACCAACTCAGGACATAGCGATT	AACTACACATGAAGTCTTCCACCAG
MEK1	TCGACTCCATGGCCAACTCCTT	TCCACCTGGCACCCAAACATC
Rac1	TGCCGATGTGTTCTTAATTTGC	CTTCTTCTCCTTCAGTTTCTCGATC
E2F1	CCGTGGACTCTTCGGAGAACTTT	GGTGGTGACACTATGGTGGCAG
Cdk4	GAGCATCCCAATGTTGTCCG	CAGATATGTCCTTAGGTCCTGGTCTA
CDC42	GGCGATGGTGCTGTTGGTAA	GCGGTCGTAATCTGTCATAATCCT
β-actin	CTGAAGTACCCCATCGAGCAC	ATAGCACAGCCTGGATAGCAAC
mir-34a-5p	TGGCAGTGTCTTAGCTGGTTGT	
mir-195-5p	TAGCAGCACAGAAATATTGGC	
mir-30c-5p	GTAAACATCCTACACTCTCAGC	
mir-130b-3p	CAGTGCAATGATGAAAGGGC	
U6	CTCGCTTCGGCAGCACA	AACGCTTCACGAATTTGCGT

### Western blot

Proteins were extracted measured with western blots as previously described [36–38]. Cells that had been treated with 50 μM DHA or vehicle were harvested, washed twice in ice-cold PBS, sonicated in RIPA buffer (Beyotime Institute of Biotechnology, Beijing, China), and homogenized. The debris was removed by centrifugation at 12,000 g for 10 min at 4°C, and protein concentrations were determined using the BCA Protein assay according to the manufacturer's instructions. Samples containing equal amounts of protein (50 μg) were separated by electrophoresis on 10% or 15% polyacrylamide SDS gels (100 V for 1 to 2 hours) and transferred to polyvinylidene difluoride (PVDF) membranes by electroblotting (100 V for 1 hour at 4°C). Running and transfer times and voltages were altered slightly as needed to optimize results. The membranes were then blocked by incubation with 5% skim milk in TBST buffer (TBS plus 0.1% Tween 20) for 2 hours and then incubated with the appropriate primary antibody overnight at 4°C with gentle agitation. The membranes were then washed several times and incubated with the appropriate horseradish peroxidase-conjugated secondary antibody (Santa Cruz Biotechnology, Carlsbad, CA, USA) for 1 hour at room temperature. Finally, membranes were washed again and protein bands were visualized with an enhanced chemiluminescence (ECL) kit, followed by exposure to X-ray film. β-actin was simultaneously measured as a loading control.

### Transfection of microRNAs

miR-34a-5p, miR-195-5p, miR-30c-5p, miR-130b-3p, mir-34a-5p specific inhibitor (miR-34a-5p antisense oligodeoxyribonucleotide, AMO-34a-5p), mir-195-5p specific inhibitor (miR-195-5p antisense oligodeoxyribonucleotide, AMO-195-5p), mir-30c-5p specific inhibitor (miR-30c-5p antisense oligodeoxyribonucleotide, AMO-30c-5p), mir-130b-3p specific inhibitor (miR-130b-3p antisense oligodeoxyribonucleotide, AMO-130b-3p), and negative control miRNA (NC) were obtained from RiboBio (Guangzhou, China). Pancreatic cancer cells at 70-80% confluence were transfected with each microRNA using the riboFect^TM^ CP transfection kit (RiboBio) according to manufacturer's instructions. 48 h after transfection, the protein levels of mRNAs targeted by the microRNAs, including Cdk4, Cdk6, VEGF, IKKα, MEK1, E2F3, Rac1, E2F1, and CDC42, were detected by western blot.

### Animal tumor model and treatments

Female nude BALB/c mice (4-6 weeks old) were purchased from the Animal Research Center at The First Clinical Medical School of Harbin Medical University (Harbin, China). All animal procedures were approved and reviewed by the Animal Care and Use Committee of The First Clinical Medical School of Harbin Medical University. BxPC-3 cells were used for *in vivo* experiments. 5×10^6^ cells were subcutaneously injected into the flanks of mice to establish tumors. Tumor sizes were measured every three days with calipers, and volumes were estimated according to the formula V=(π/6)×(larger diameter)×(smaller diameter)^2^. When tumors reached approximately 120 mm^3^, mice were randomly assigned to one of two groups (five mice per group): the control group (once daily i.p. injection of DMSO dissolved in 200 μL PBS) or the DHA-treated group (10 mg/kg, once daily by i.p. injection). The DHA dose used in this experiment was selected based on preliminary experiments and a previous study [17]. Mice were closely monitored. After eighteen days, all mice were euthanized and tumors were excised. Each tumor was divided in two: the first tumor sample was fixed with 10% buffered formalin, and the second was stored at −80% for further analysis.

### Ki-67 immunohistochemistry

Briefly, paraffin-embedded tissue sections (5 μm) were immunostained with an anti-Ki-67 antibody as previously described [36, 39]. Numbers of Ki-67-positive cells were counted in ten randomly-selected microscopic fields at ×400 magnification.

### Quantification of apoptosis in tumor sections

Apoptosis was quantified as previously described [36, 39]. Briefly, paraffin-embedded tissue sections (5 μm) were stained with the TUNEL agent (Roche, Shanghai, China), and numbers of TUNEL-positive cells were counted in ten randomly selected microscopic fields at ×400 magnification.

### Tumor microvessel density

Tumor microvessel densities were quantified as previously described [36, 39]. Briefly, tumor sections were immunostained with anti-CD31 antobody, numbers of microvessels were counted in randomly selected ten microscopic fields at ×200 magnification, and microvessel densities were calculated.

### Statistical analysis

Results are expressed as means ± standard deviations (SD). The significance of differences between histopathologic scores was assessed using the Kruskal–CWallis test. Continuous data were analyzed by ANOVA and the Student–CNewman–CKeuls test. The hypergeometric test was used to identify significantly enriched pathways. Differences were considered statistically significant when *p*<0.05. All statistical analyses were performed using the R 3.1.3 framework.

## SUPPLEMENTARY FIGURES


