# Supplementary material for: Dihydroartemisinin suppresses pancreatic cancer cells via a microRNA-mRNA regulatory network
Source: Oncotarget. 2016 Aug 23;7(38):62460–73. doi: 10.18632/oncotarget.11517 (PMC5308739; doi:10.18632/oncotarget.11517)
Supplement: Supplementary file 1 [file oncotarget-07-62460-s001.pdf]

Dihydroartemisinin suppresses pancreatic cancer cells via a microRNA-mRNA regulatory network

SUPPLEMENTARY FIGURES

Confirmation of levels of miRNAs identified by microarray and systematic analysis in PANC-1, BxPC-3, and HPDE6-C7 cells with or without DHA treatment

As shown in Supplementary Figure S2, DHA treatment slightly up-regulated miR-34a-5p, miR-195-5p, miR-30c-5p, and miR-130b-3p expression compared to the group of HPDE6-C7. The expression of miR-34a-5p miR-195-5p, miR-30c-5p and miR-130b-3p are significantly in a low level in PANC-1 and BxPC-3 cell lines compared to HPDE6-C7 cell line.

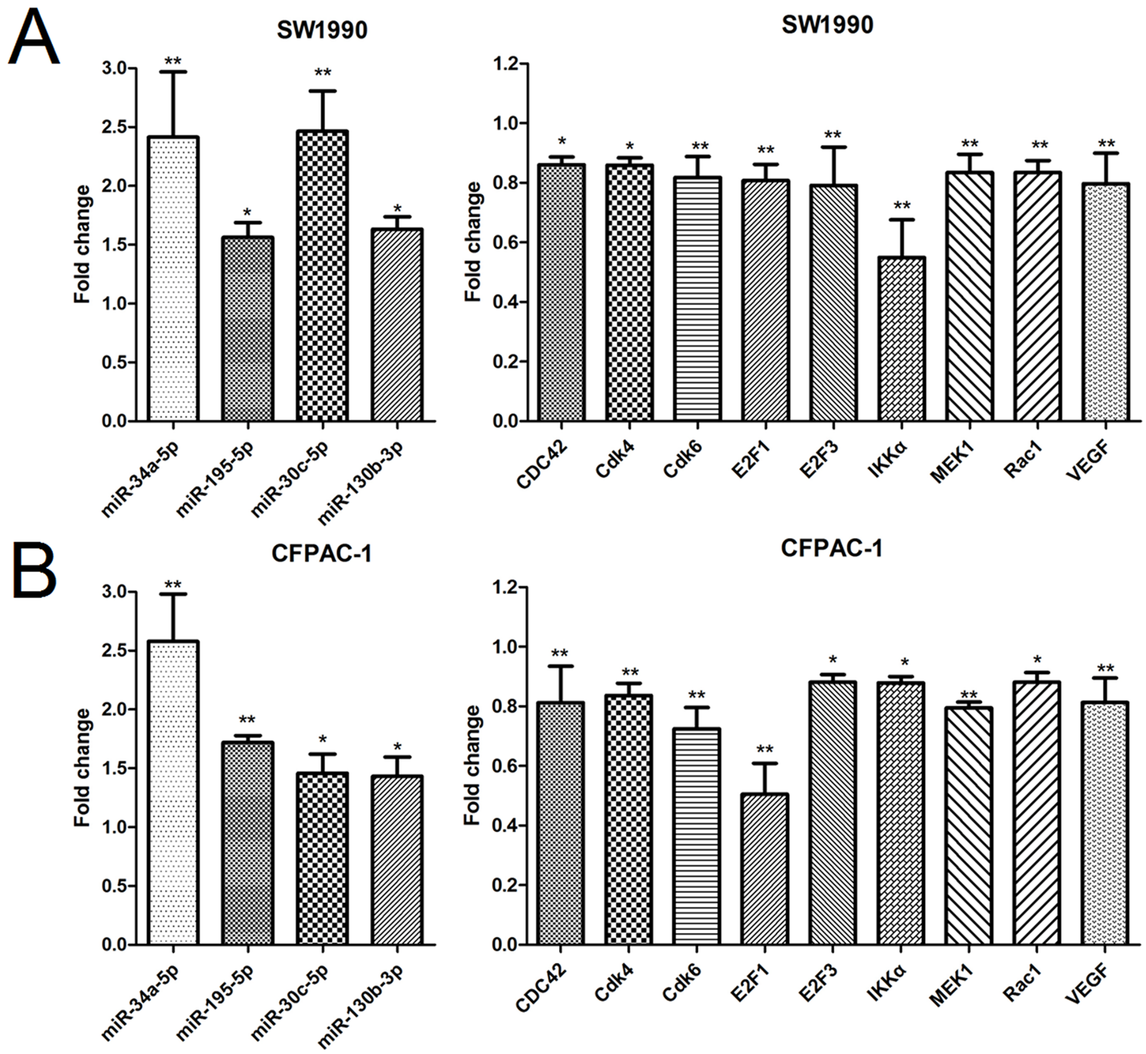

Supplementary Figure S1: Confirmation of the 4 microRNAs and their 9 mRNA targets in SW1990 and CFPAC-1 cells by qRT-PCR. A. Differential expression of the 4 microRNAs and their 9 mRNA targets in SW1990 cells. *p<0.05, **p<0.01, compared to the control. **B.** Differential expression of the 4 microRNAs and their 9 mRNA targets in CFPAC-1 cells. *p<0.05, **p<0.01, compared to the control.

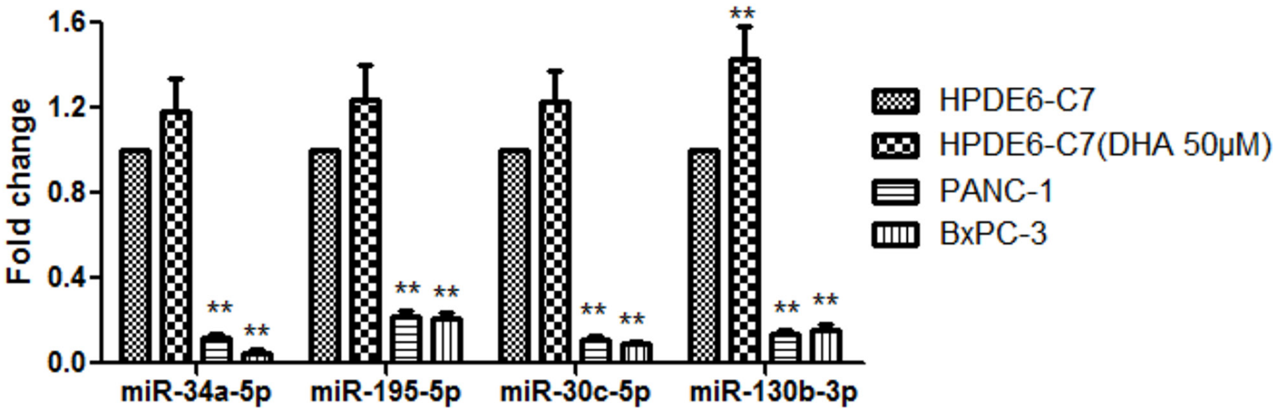

Supplementary Figure S2: Confirmation of levels of miRNAs identified by microarray and systematic analysis in PANC-1, BxPC-3, and HPDE6-C7 cells with or without DHA treatment. DHA treatment slightly upregulated miR-34a-5p, miR-195-5p, miR-30c-5p, and miR-130b-3p expression in HPDE6-C7 cells. The expression of miR-34a-5p miR-195-5p, miR-30c-5p and miR-130b-3p are significantly in a low level in PANC-1 and BxPC-3 cell lines compared to HPDE6-C7 cell line.

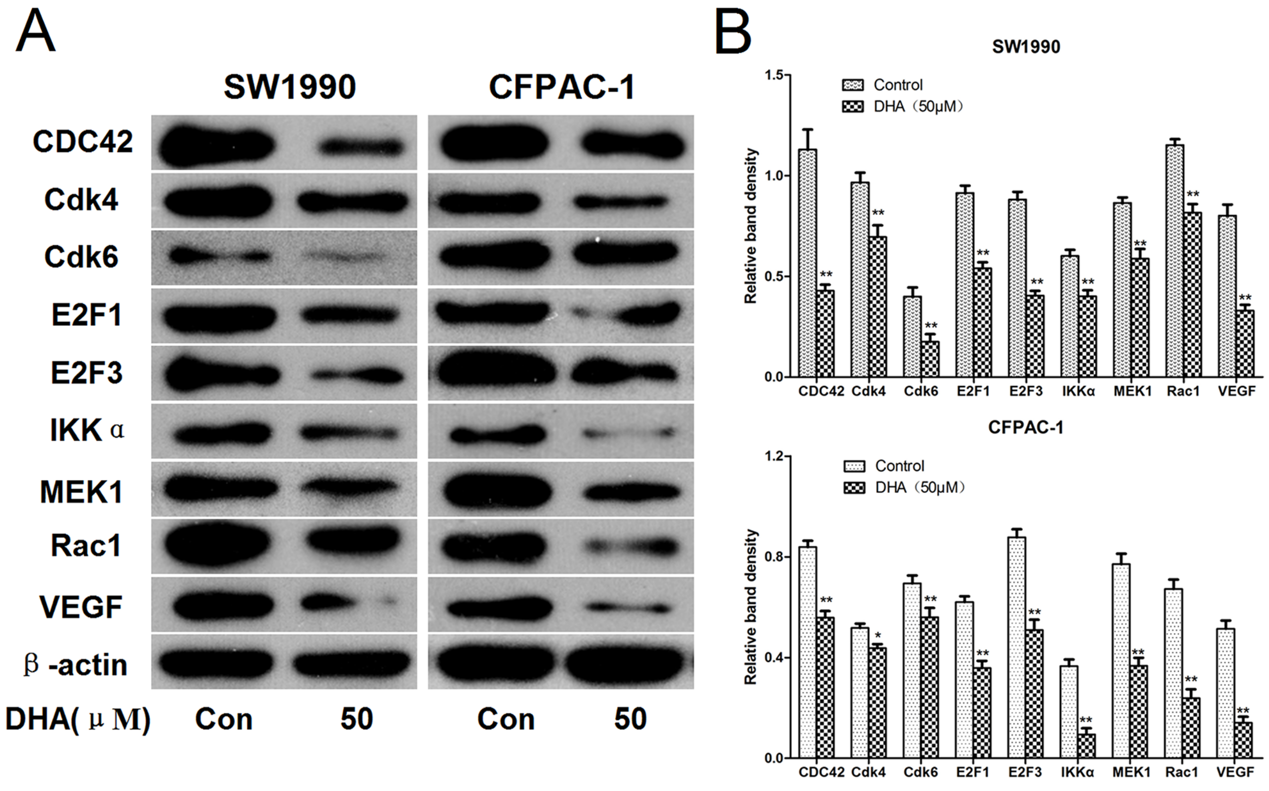

Supplementary Figure S3: Confirmation of protein levels translated from the relevant mRNAs in SW1990 and CFPAC-1 cells by western blot. A. SW1990 and CFPAC-1 pancreatic cancer cells were treated with DHA (50 μM) or vehicle for 72 h, and protein extracts were measured by western blot. β-actin was used as a protein loading control. **B.** The density of each band was measured and compared to β-actin. *p<0.05, **p<0.01, compared to the control.

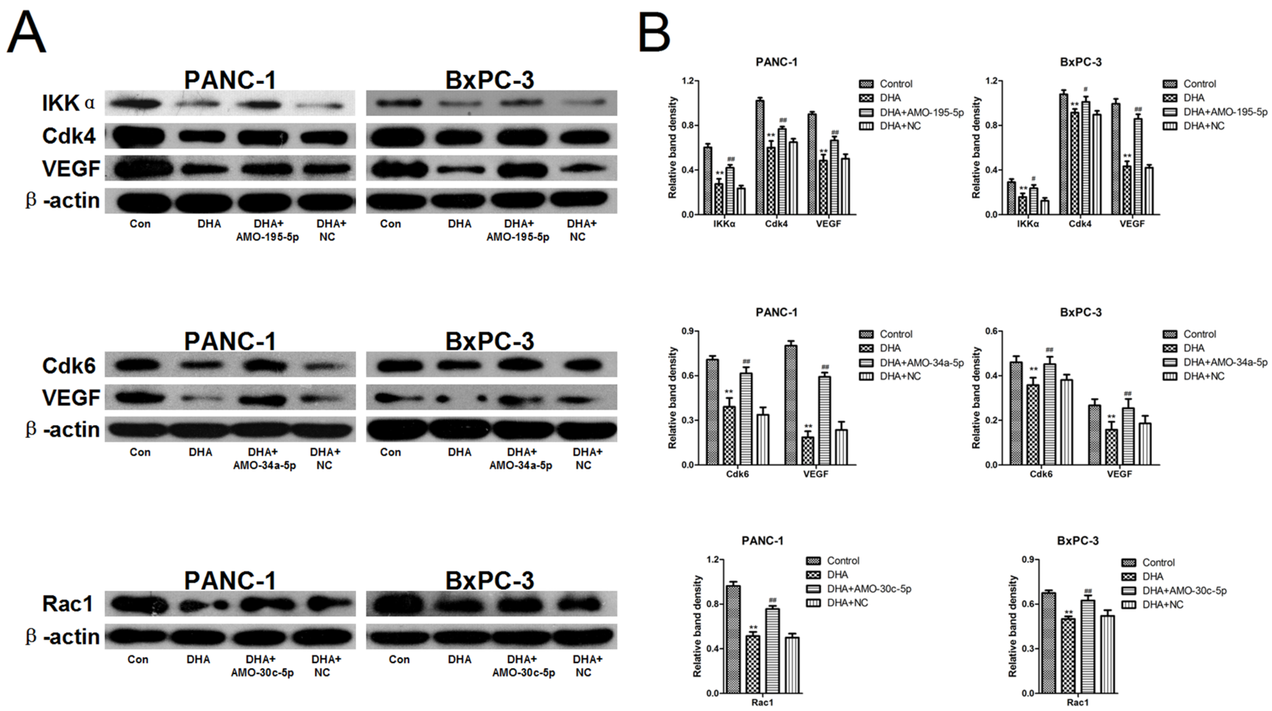

Supplementary Figure S4: Confirmation of the anti-pancreatic cancer effects of DHA via microRNAs identified by microarray and systematic analysis. A. PANC-1 and BxPC-3 pancreatic cancer cells were transfected with negative control miRNA, miR-34a-5p-specific inhibitor, miR-195-5p-specific inhibitor, or miR-30c-5p-specific inhibitor. After 24 h, these cells were treated with DHA (50 μM) or vehicle for 48 h and protein extracts were measured by western blot. β-actin was used as a protein loading control. **B.** The density of each band was measured and compared to β-actin. **p*<0.05, ***p*<0.01, compared to the control; #*p*<0.05, ##*p*<0.01 compared to the DHA-treated group.
